# FAITH, HOPE, AND LOVE: UNDERSTANDING RELIGIOSITY AND HOPE AMONG OLDER AFRICAN AMERICAN COUPLES

**DOI:** 10.1093/geroni/igac059.2430

**Published:** 2022-12-20

**Authors:** Antonius Skipper, Andrew Rose

**Affiliations:** Georgia State University, Atlanta, Georgia, United States; Texas Tech University, Lubbock, Texas, United States

## Abstract

Older African Americans frequently turn to their religion or spiritual faith as a source of coping and resilience. Disproportionately burdened by numerous disparities and stressors, many African American utilize religion as a source hope for the future. Existing studies suggest that higher levels of positive religious coping are associated with higher levels of hope, and more frequent experiences with negative religious coping are associated with lower levels of hope. However, the relationship between religious coping and hope is underexamined among one of the most religious dyads in the U.S., older African American couples. This study utilizes data from 194 older African American couples (146 married and 48 cohabiting), with each partner between the age of 50 and 86 years, to examine the dyadic relationship between religious coping and hope. Actor Partner Interdependence Models revealed that men’s religious coping was associated with their own hope, and women’s religious coping was associated with their own hope. One unexpected partner effect was identified and found that women’s positive religious coping was negatively associated with men’s hope. Given the dearth of research on older African American couples, along with the need to better understand religious significance in psychosocial dyadic outcomes, this study offers several implications for engaging African American couples in relational counseling and therapy across the life course.

